# Effect of design geometry, exposure energy, cytophilic molecules, cell type and load in fabrication of single-cell arrays using micro-contact printing

**DOI:** 10.1038/s41598-020-72080-w

**Published:** 2020-09-16

**Authors:** Swapnil Vilas Bhujbal, Maren Dekov, Vegar Ottesen, Karen Dunker, Rahmi Lale, Marit Sletmoen

**Affiliations:** 1 Department of Biotechnology and Food Science, Norwegian University of Science and Technology, 7491 Trondheim, Norway; 2 Department of Chemical Engineering, Norwegian University of Science and Technology, 7491 Trondheim, Norway

**Keywords:** Soft lithography, Nanofabrication and nanopatterning

## Abstract

In this study a range of factors influencing the fabrication of single-cell arrays (SCAs) are identified and investigated. Micro-contact printing was used to introduce spots coated with polyethyleneimine or Matrigel on glass surfaces pre-coated with polyethylene glycol. Unmodified *E. coli*, *Synechococcus* sp., *Chlamydomonas reinhardtii* as well as diverse mammalian cells including HUVEC, AAV293, U87, OHS, PC3, SW480, HepG2 and AY-27 were successfully immobilised onto the chemically coated spots. The developed SCAs show high cell viability and probability for capturing single-cells. A discrepancy between the size and shape of the squares described in the design file and the actual structures obtained in the final PDMS structure is characterised and quantified. The discrepancy is found to be depending on the exposure energy used in the photolithography process as well as the size of the squares and their separation distance as detailed in the design file. In addition to these factors, the effect of the cell density loaded onto the patterned surfaces is also characterised. The systematic characterisation of key parameters that need to be optimised prior to the fabrication of SCAs is essential in order to increase the efficiency and reproducibility of future fabrication of SCAs for single-cell studies.

## Introduction

**Figure 1:**
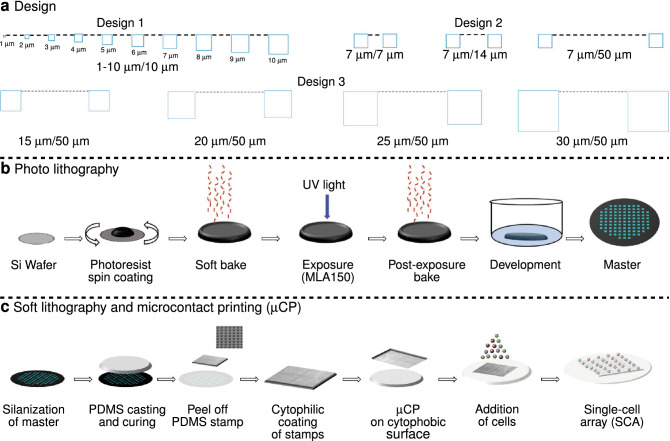
Graphical representation of steps that must be optimised when aiming at fabricating a single-cell array (SCA). (**a**) A design file is made in a layout editor software (e.g. CleWin or AutoCAD) with appropriate size (width (W)) and separation distance (D) between consecutive geometric shapes. The figure presents the different designs that were used in the current study. Design 1 consists of squares from 1 to $10\,\upmu \hbox {m}$ in width separated by $10\,\upmu \hbox {m}$. Design 2 consists of squares of $7\,\upmu \hbox {m}$ separated by either 7, 14 or $50\,\upmu \hbox {m}$. Design 3 consists of squares of either 15, 20, 25 and $30\,\upmu \hbox {m}$ separated by $50\,\upmu \hbox {m}$. All designs in (**a**) are shown top-down. (**b**) The figure presents the steps involved in maskless photolithography. Maskless photolithography directly transfers information from design files onto the photoresist film using UV light and eliminates the need to create an expensive physical photomask. The fabricated wafer is named master, and is further used for soft lithography. (**c**) The figure presents the steps involved in soft lithography and micro-contact printing ($\upmu \hbox {CP}$). The PDMS stamp is coated with cytophilic molecules and is further processed for $\upmu \hbox {CP}$ in order to obtain SCAs.

Much of our knowledge about cellular behaviour is based on cell population averages. However, all cellular systems are fundamentally heterogeneous in the sense that cellular behaviour varies between individual cells within a population which can also change over time. This gives rise to various sub-populations of cells exhibiting different and potentially important characteristics that are often not visible if studying cellular behaviour at the population level^1^. This heterogeneity may arise for a number of different reasons including improved population survival and functionality^2,3^. The study of population heterogeneity has therefore become a central topic within life sciences and resulted in several different approaches to obtain and study arrays of immobilised single cells^4^. These approaches are faced with multiple challenges such as they must permit simultaneous study of a high number of cells, provide resolution at the single-cell level while maintaining cell viability and function. One of the proposed technologies is based on micro-contact printing ($\upmu \hbox {CP}$). $\upmu \hbox {CP}$ can be used to immobilise cells in an ordered array and, if combined with microscopy, monitor dynamic changes in cellular activity without compromising the overall viability and function of the cells^5^. The principle underlying the fabrication of cellular arrays using $\upmu \hbox {CP}$ is the introduction of both cytophilic (cell-friendly) and cytophobic (cell-repelling) regions by modifying the surface chemistry of a substrate. Previous studies in which $\upmu \hbox {CP}$ was used focus on how the cells interact with the substrate^6,7^. However, these studies do not provide the important details concerning the various factors affecting the $\upmu \hbox {CP}$ process. These factors include but are not limited to the energy of the radiation used to expose chosen areas of the photoresist film, the properties of the cytophilic and cytophobic coatings that are added to the glass surface and the cell load. Unsufficient attention to these factors might lead to challenges in the fabrication and reproducibility of the SCAs. Hence, the lack of systematic quantification and documentation of these factors hamper the effective use of $\upmu \hbox {CP}$ in the biological sciences. The key steps involved in $\upmu \hbox {CP}$ are: (1) fabrication of stamps with the desired geometric size and shape using photolithography^8–10^ and soft lithography^11,12^, (2) coating of the stamp with cytophilic molecules for immobilisation of cells, and (3) transfer of the cytophilic molecules onto the cytophobic substrate^13,14^. Figure 1 provides a graphical overview of the process in which $\upmu \hbox {CP}$ is used to fabricate a SCA. Photolithography makes use of a (UV) light sensitive material (photoresist) to transfer pre-defined patterns of geometric shapes to a substrate (Fig. 1b). Silicon wafers are the most commonly used substrate. A uniform photoresist coating of desired height is applied to the substrate by spin-coating. This photoresist will become either soluble (positive photoresist) or insoluble (negative photoresist) if exposed to a certain dose of light of a given wavelength^8,9^. By controlling what areas are exposed, a pattern can be created. Since a beam of light is used to deposit the energy, the maximum resolution that can be obtained will be diffraction limited. The optimal wavelength of the exposure light will be different for different photoresists, and is usually indicated in the instruction manual provided by its manufacturer. The optimal exposure dose depends on the height (H), width (W) and separation distance (D) between consecutive geometric shapes. The height of the photoresist layer depends on the speed at which the photoresist is spun on the substrate as well as the viscosity of the photoresist. The height of the photoresist layer determines the maximal height of the structures that can be obtained. The size, shape and separation distance between the geometric shapes defined in the design file should be chosen based on the knowledge concerning the final application of the patterned surfaces. The silicon substrate fabricated by photolithography is called “master” and is further used for soft lithography.

Soft lithography is a complementary extension of photolithography used for replicating a design obtained through photolithography (Fig. 1c)^12^. It is called “soft” because it uses elastomeric materials to perform the lithography. The most commonly used material is polydimethylsiloxane (PDMS). Briefly, PDMS is cast on a master and allowed to cure. After curing, the PDMS is peeled off from the master to obtain the stamps. These stamps contain patterns of geometric shapes as defined in the design file. Throughout the text, the geometric shapes present on the PDMS stamps will be referred to as pillars. To avoid deformations of pillars on the stamps, the H/W ratio should be between 0.5–5 and the H/D ratio above 0.05^15–17^. $\upmu \hbox {CP}$ is performed by coating the pillars with a chemical of interest (usually referred to as “inking”) and thereafter carefully placing the stamp on the surface which is to be patterned. Throughout the text, the imprinted pillar areas present on the surface will be referred to as spots. When aiming at fabricating cellular arrays, the pillars of the PDMS stamp are often coated with cytophilic molecules such as polydopamine (PD)^18^, polyethyleneimine (PEI)^19^, fibronectin^20^, laminin^21^ that are transferred onto a cytophobic surface like for example a surface coated with poly (L-lysine) polymers with poly (ethylene glycol) side-chains (PLL-PEG), to obtain an array of cytophilic spots. Cells are then seeded on these patterned surface, leading to an array of immobilised single cells.

The present study was undertaken to investigate and document the effect of exposure energy using Maskless Aligner MLA-150 on the negative photoresist microresist-direct write laser (mr-DWL). The exposure energy is evaluated as a function of the dimensions of the geometric shapes that are to be transferred to the photoresist layer. Furthermore, the important relationship between cell size, cell load, and the width and separation distance between the cytophilic surface spots required to immobilise single cells using $\upmu \hbox {CP}$ is addressed. The current study reports the fabrication of SCAs of *Escherichia coli*, *Synechococcus* and *Chlamydomonas reinhardtii* through $\upmu \hbox {CP}$ based deposition of the cytophilic chemical PEI. The study also reports the fabrication of SCAs of chosen mammalian cells including HUVEC, AAV-293, U87,OHS,PC3, SW480, Hep-G2, AY-27 using a protein mixture with the trade name Matrigel. To our best knowledge, this is the first study which covers a holistic view of the factors influencing fabrication of SCA using $\upmu \hbox {CP}$.

## Results

### PDMS pillar width depends on UV light exposure energy

**Figure 2:**
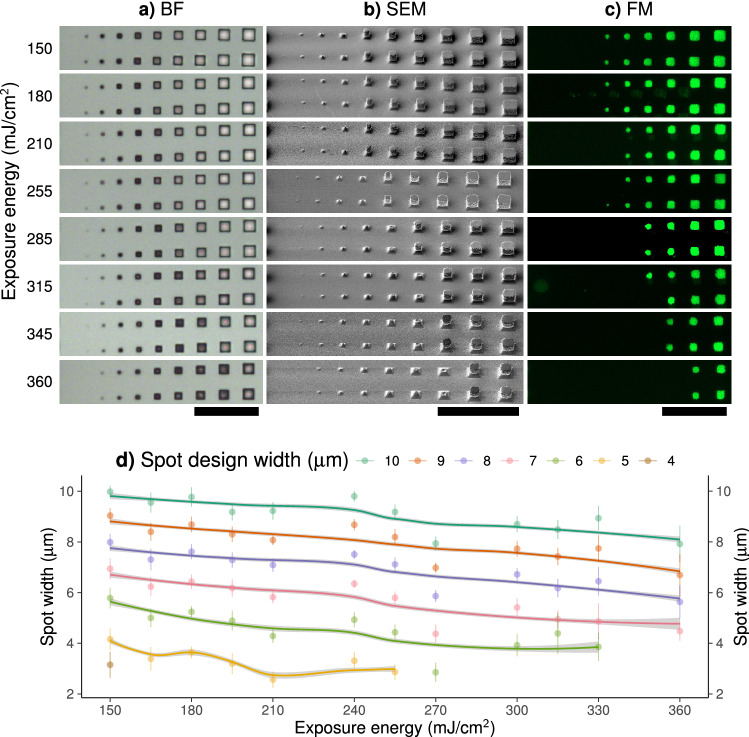
Relationship between the energy of the UV light used during exposure of the photoresist and the size of the square shaped pillars present on the PDMS stamp. The master and stamps used in the experiment were fabricated based on design 1 (Fig. 1a) and had dimensions in the range 1–10 $\upmu \hbox {m}$ with 10 $\upmu \hbox {m}$ horizontal spacing and were made using the mr-DWL5 photoresist. (**a**) and (**b**) Images of the PDMS stamps obtained using bright field (BF) light microscopy (**a**), and scanning electron microscopy (SEM) (**b**), respectively. The SEM images were recorded at 45$^{\circ }$ tilt. (**c**) Fluorescence microscopy image of a glass surface onto which PLL-FITC had been deposited using a PDMS stamp fabricated as described above. Scale bars are shown as black lines under each image column, and all scale bars correspond to a distance of 50 $\upmu \hbox {m}$. (**d**) Width of spots from fluorescence microscopy plotted against exposure energy. Continuous lines included in the figure reflect the loess-regression lines for each spot size. The ribbon around each line shows the 95% confidence interval. Points show mean widths and error bars show standard deviation.

A first aim of this study was to identify the optimal UV light exposure energy for the fabrication of design 1 using the mr-DWL 5 photoresist. In order to reach this aim, we applied UV light exposure energies ranging from 150 to $\hbox {360 mJ/}\hbox {cm}^2$, with a step size of $15\hbox { mJ/}\hbox {cm}^2$. Bright-field (BF) microscopy imaging of the masters revealed that the hollow square (will hereafter be referred to as wells) designed to have a width equal to 1 $\upmu \hbox {m}$ (Fig. 1a, design 1) were not properly transferred to the master irrespective of the exposure energy used. The well designed to have a width equal to $2\,\upmu \hbox {m}$ was not visible if applying exposure energies beyond $345\ \hbox {mJ/cm}^2$ (Fig. 2a). Wells of expected width below $5\,\upmu \hbox {m}$ appeared rounded in the BF images, indicating that these areas were overexposed. The larger wells appeared to have a square shape, indicating that the exposure was successful. As the exposure energy increased, all wells appeared rounded.

SEM images of PDMS stamps revealed the relationship between exposure energy and PDMS pillar width. As the exposure energy increased, an increasing number of pillars were lost, and the remaining pillars were characterised by a broad edge base and rounded top. The pillars with expected widths of 1-$4\,\upmu \hbox {m}$ were missing on all of the PDMS stamps, irrespective of exposure energy. The pillars with dimensions of $5\,\upmu \hbox {m}$ were missing if exposure energies beyond $240\hbox { mJ/cm}^2$ had been used. Exposure energies above $\hbox {315 mJ/}\hbox {cm}^2$ lead to the disappearance of pillars with an expected width of less than $7\,\upmu \hbox {m}$. Only the pillars of expected width ranging from $8\,\upmu \hbox {m}$ to 10 $\upmu \hbox {m}$ are seen when applying an exposure energy equal to $\hbox {360 mJ/}\hbox {cm}^2$ (see Fig. 2).

In order to reveal the shape and size of the top surface of the pillar, i.e. the part of the pillar that comes in contact with the glass surface during $\upmu \hbox {CP}$, PDMS stamps made based on design 1 were coated with poly-L-lysine grafted with Fluorescein isothiocyanate (PLL-FITC). Fluorescence microscopy was used to reveal the imprint deposited on glass slides (Fig. 2c). The results obtained show good correspondence with the SEM results in the sense that the shape, size, and number of FITC-coated spots are similar to the pillars observed in the SEM images (Fig. 2b). The area of each FITC-coated spot was calculated using FIJI. Figure 2d presents the spot widths as a function of exposure energy. In the figure images of stamps and imprinted surfaces obtained for eight chosen exposure energies are presented. The remaining images underlying the data shown in (Fig. 2d) are presented in Supplementary Figure S1. As the exposure energy increases, the width of spots is reduced, reflecting the decreased width of the upper part of the PDMS pillar. The reduced spot width also leads to increased spacing between the FITC spots compared to the separation distance (D) given in the design file.

### Importance of PDMS pillar width and separation distance for the size of the deposited surface spots

**Figure 3:**
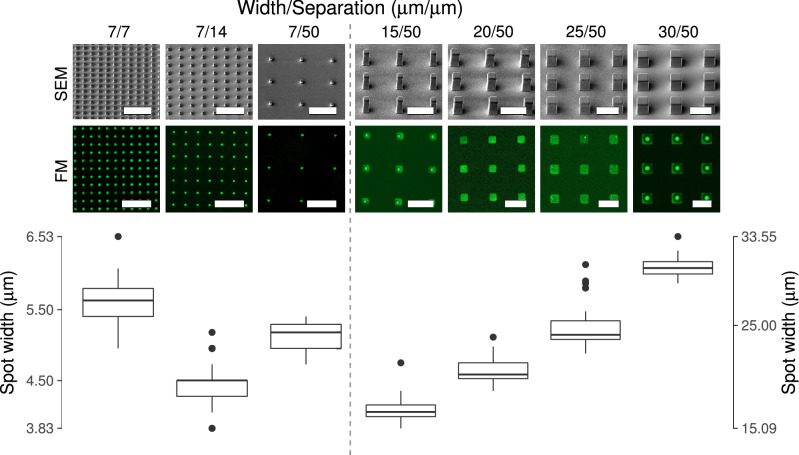
Effect of width (W) and separating distance (D) of PDMS pillars on the size of the FITC coated surface spots. The results are shown as a function of pillar separation distance and as a function of pillar width. The dimensions of the PDMS pillars are revealed in the SEM images and the size of the FITC surface spots could be determined based on fluorescence microscopy (FM) images. All scale bars represent a distance equal to 50 $\upmu \hbox {m}$. Tukey box-plots show the width of the FITC coated surface spots as determined for the FM micrographs. The annotation above each column of images and related Tukey box-plot refers to the dimensions expected based on the design file. For 7, 14 and 50 $\upmu \hbox {m}$ expected pillar separation, 139, 176 and 34 spot imprints were inspected, respectively. The determination of the width of each of these imprints forms the basis for the box plots. For stamps with an expected pillar separation distance equal to 50 $\upmu \hbox {m}$, but expected pillar width increasing with 5 $\upmu \hbox {m}$ steps in the range 15 $\upmu \hbox {m}$ till 30 $\upmu \hbox {m}$, 74, 51, 52 and 44 spots were measured, respectively.

The surface of the PDMS pillars were visualised using SEM. The SEM images revealed that the pillars on the PDMS stamp obtained based on design 2 were of uniform shape and size with minor undercut edge base and rounded surface at top (Fig. 3).

The pillars on the PDMS stamps made based on design 3 were uniform with vertical edge base as shown in Fig. 3. However, the pillars present in the 15 $\upmu \hbox {m}/50\ \upmu \hbox {m}$ (where the first number refers to the length of the side of the square shaped pillar and the second number is the separation distance between pillars) and $20\,\upmu \hbox {m}/50\ \upmu \hbox {m}$ showed a rounded pillar surface. The pillars with dimensions $25\,\upmu \hbox {m}/50\ \upmu \hbox {m}$ and $30\,\upmu \hbox {m}/50\ \upmu \hbox {m}$ showed a flat pillar surface with sharp well-defined edges.

However, quantification of the size of the PLL-FITC imprinted spots showed that the imprinted spot was smaller than expected based on the design file (Table 1). For pillars with the expected side length of $7\,\upmu \hbox {m}$ the average length varied from 4.1 to $3.9\,\upmu \hbox {m}$ with increasing separation distance between the pillars. For pillars of design 3, as design width increased, the actual width observed approached the expected width, and for the design with expected width $30\,\upmu \hbox {m}$ the measured width was $30.6\,\upmu \hbox {m}$.

**Table 1:** Comparison between the size of the FITC coated spots and the inter spot distances as determined based on image analysis and the spot sizes and inter spot distances expected based on the design file.

Design width	Actual width	Design distance	Actual distance
7.0	4.1	7.0	9.9
7.0	3.9	14.0	17.1
7.0	3.9	50.0	53.1
15.0	13.5	50.0	51.6
20.0	16.2	50.0	53.9
25.0	21.4	50.0	53.6
30.0	30.6	50.0	49.4

All values are in $\upmu \hbox {m}$.

### Importance of spot-size, inter-spot distance as well as cell load for the regularity of the cell array

**Figure 4:**
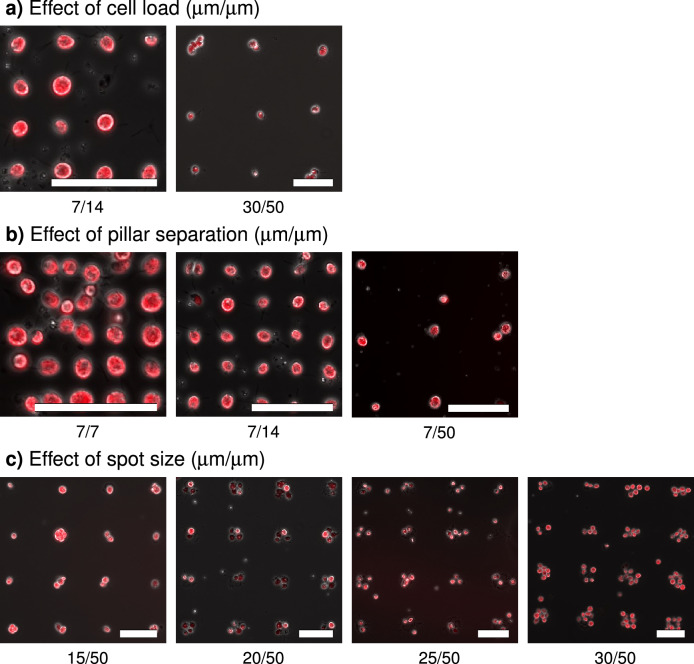
Relationship between cell load, pillar separation and spot size. *Chlamydomonas reinhardtii* (cell width 7–10 $\upmu \hbox {m}$) were immobilised on imprints of PEI made with stamps of design 2 and 3. (**a**) Effect of cell load—if cell load is less than the total area of the imprinted array, then the probability of obtaining empty spots on the array increases ($7\ \upmu \hbox {m}/14\,\upmu \hbox {m}$). However, the probability of immobilising single cells on an array with larger spot size increases ($30\,\upmu \hbox {m}/50\,\upmu \hbox {m}$). (**b**) Effect of inter pillar spacing—cells immobilised on $7\,\upmu \hbox {m}/7\,\upmu \hbox {m}$ are not as well separated as cells immobilised on 7 $\upmu \hbox {m}$/14 $\upmu \hbox {m}$ and $7\,\upmu \hbox {m}/50\,\upmu \hbox {m}$. (**c**) Effect of spot size—if cell width is smaller than the imprinted spot width then the number of cells immobilised on the array increases. The spot width of $15\,\upmu \hbox {m}/50\,\upmu \hbox {m}$, $20\,\upmu \hbox {m}/50\,\upmu \hbox {m}$, $25\,\upmu \hbox {m}/50\,\upmu \hbox {m}$, and $30\,\upmu \hbox {m}/\,50\upmu \hbox {m}$ had approximately 2, 3, 5 and 7 cells per imprinted spot on the array respectively. 5(6)-Carboxynaphthofluorescein (CNF) fluorescence confirms the viability of the cells. All images were taken at 20$\times$ magnification. Scale bars represent a distance equal to 50 $\upmu \hbox {m}$.

To obtain a single cell in each predefined surface spot the imprinted spot size as well as the spacing between the consecutive spots must be optimised to fit both the cell size and the cell load. In the current study, *Chlamydomonas reinhardtii* cells (cell width 7–10 $\upmu \hbox {m}$) were immobilised onto cytophilic PEI functionalized spots on cytophobic PEGylated glass slides. Figure 4a) presents the effect of the cell load, added to the array. As the cell load decreases, the probability of observing empty (no cells immobilised) spots on the array increases (Fig. 4a, 7 $\upmu \hbox {m}/14\ \upmu \hbox {m}$). On the surfaces stamped with the 7 $\upmu \hbox {m}/7\ \upmu \hbox {m}$ pillars, cells were observed to form bridges between the cells immobilised onto the cytophilic spots, most likely due to cell-to-cell adhesion (Fig. 4b). These additional cells reduces the regularity of the SCA. When increasing the inter-spot separation distance to 14 $\upmu \hbox {m}$ this adhesion mechanism becomes less likely and a more regular SCA is obtained (Fig. 4b). The size of the cytophilic spots is also important. In order to obtain a SCA the spot size should be smaller than the diameter of the cell (Fig. 4b). The spot width of 15 $\upmu \hbox {m}/50\,\upmu \hbox {m}$, $20\ \upmu \hbox {m}/50\ \upmu \hbox {m}$, $25\ \upmu \hbox {m}/50\ \upmu \hbox {m}$, and $30\ \upmu \hbox {m}/50\ \upmu \hbox {m}$ had approximately 2, 3, 5 and 7 cells per spot on the array, respectively.

### Immobilisation of *E. coli*, *Synechococcus* and diverse mammalian cells

**Figure 5:**
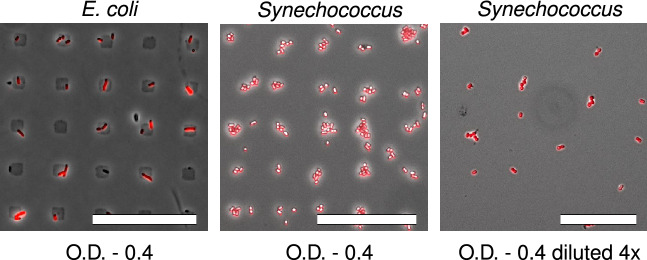
*E. coli* (cell width 2–3 $\upmu \hbox {m}$) and *Synechococcus* cells (cell width 0.8–1.5 $\upmu \hbox {m}$) are effectively immobilised using PEI and a spot size of $7\,\upmu \hbox {m}/14\,\upmu \hbox {m}$. At O.D. 0.4 the probability of single imobilised cells per spot is higher for *E. coli* than *Synechococcus*. When the density of *Synechococcus* cells is diluted of its initial value the probability of obtaining single cells per spot increases, however, some of the spots had no cell immobilised on it. mCherry and CNF fluorescence confirms the viability of *E. coli* and *Synechococcus* cells, respectively. The magnification is 20 $\times$ and the scale bars represent a distance equal to 50 $\upmu \hbox {m}$.

When aiming at fabricating a SCA, the choice of adhesion-promoting chemical must be optimised for each cell type. Our results demonstrate that Gram-negative bacteria *E.coli* (cell width 2–$3\,\upmu \hbox {m}$) and *Synechococcus* (cell width 0.8–1.5 $\upmu \hbox {m}$) can be successfully immobilised on PEI functionalized spots on PEGylated glass slides (Fig. 5). Most of the PEI coated spots were occupied by single *E.coli* cells. In a first experimental series the functionalized glass surfaces were covered with a log phase culture of *Synechococcus* cells characterised by OD equal to 0.4. Under these experimental conditions multiple cells were observed per spot. When the *Synechococcus* cell density was reduced to 25% of its initial value, most of the spots on the array were occupied by single cells. mCherry and CNF fluorescence confirms the viability of *E. coli* and *Synechococcus* cells, respectively.

**Figure 6:**
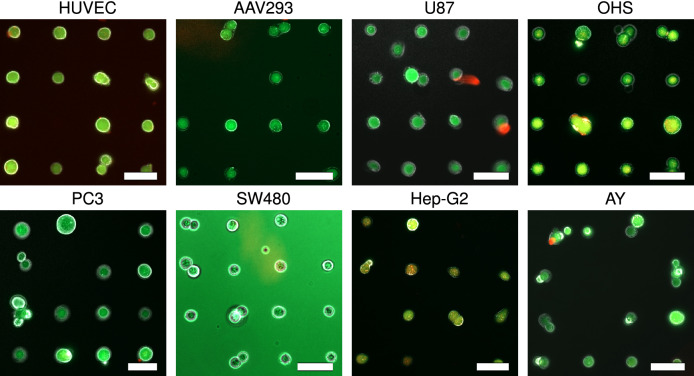
Mammalian cells are effectively immobilised on Matrigel coated spots with a size of $15\,\upmu \hbox {m}/50\,\upmu \hbox {m}$. Live dead staining confirms the viability of the cells (green colour represents live cells, red colour represents dead cells). Images are taken at 20 $\times$ magnification. All scale bars represent a distance equal to 50 $\upmu \hbox {m}$.

Mammalian cells were immobilised onto surface spots coated with Matrigel. The Matrigel was deposited onto the surface using PDMS pillars of $15\,\upmu \hbox {m}/50\,\upmu \hbox {m}$, leading to the cellular arrays presented in Fig. 6. Most of the spots on the array were occupied by single cells. Live dead staining confirms the viability of the cells.

## Discussion

The SCA technology described here opens for high-throughput studies of single cells on a scale barely thinkable a few decades ago. This technology is therefore a powerful tool for a variety of applications including, but not limited to, the testing of the effect of drugs, toxins or other external stimuli on cells^4^. Photolithography and soft lithography are often used for the fabrication of cellular arrays. Despite their unquestionable importance for future studies in life sciences, the effect of the parameter settings chosen when using these techniques for the fabrication of cellular arrays are under-studied. The lack of systematic studies is a challenge since even small variations in the parameter settings can have huge impact on the quality of the SCA obtained. The present study focuses on some key factors affecting the photolithography process. We used maskless photolithography, as it eliminates the need for creating an expensive photomask, and changes in design files can also be quickly implemented^22,23^. This not only saves time and money, but also provides extreme flexibility allowing researchers to quickly adjust experiments or correct potential errors. In addition, maskless photolithography provides non-contact exposure at high speed, making it the ideal tool for quick fabrication protocols. We used the mr-DWL series photoresist as an alternative to the often used SU-8 photoresist since the latter one is difficult to process with the employed maskless aligner.

The recommended exposure energy for mr-DWL 5 is in the range of 300–500 mJ/$\hbox {cm}^2$, depending on height of the features^24^. In the current work we performed a systematic study of UV light exposure energies in the range 150–360 mJ/$\hbox {cm}^2$. The SEM images obtained (Figs. 2, 3) show that when aiming to fabricate PDMS stamps with closely separated pillars, the energy of the UV light should be lower than the recommended values. This information is essential in order to obtain satisfactory results. The fluorescence microscopy images obtained for glass surfaces onto which PLL-FITC had been deposited using a PDMS stamp confirmed that the width of the pillars on the PDMS stamp are smaller than the width described in the design file. A quantitative analysis of the FM images reveal a gradually decreasing width of the pillars for gradually increasing energy of the UV light (Fig. 2). This correlation might be explained by the optical proximity effect (OPE), which explains how the shape for a 2-D pattern fabricated using lithographic tools will depend on also the proximity of nearby features^10,25–27^. For large features, the diffraction patterns of exposure do not strongly depend on the separation distance between the geometric shapes. However, as feature width decreases, the differences between closely spaced and less closely spaced shapes becomes apparent^28^. A number of parameters affect the magnitude of the OPE, including photoresist type, thickness of photoresist layer, wavelength of exposure light, exposure energy, numerical aperture, and illuminator settings^27–29^. As a consequence, the exact magnitude of OPE needs to be determined empirically and re-evaluated whenever changes in the process are implemented. Several methods that allow to reduce the OPE have been proposed^28,30–32^. One of these methods is based on a modification of the geometries of the design files to compensate for the non-ideal properties of the lithography process^33–35^.

Whereas the OPE causes severe challenges when working with the mr-DWL 5 series of photoresists, only minor OPE was observed in the PDMS stamps made from wafers filled with the photoresist mr-DWL 40 (Fig. 3b). This could be due to the different viscosity of the two photoresists, differences in the height of the photoresist layer after spin coating, or the spacing between the pillars not being sufficient. However, also when working with the mr-DWL 40 photoresist the PDMS stamps with pillar width $\le \,20\ \upmu \hbox {m}$ showed rounded surfaces at the top of the pillars. This again illustrates that the optical proximity effect becomes increasingly important as the pillar size decreases. The analysis of PLL-FITC deposited spots confirm that the pillars with expected side width of 15–$25\,\upmu \hbox {m}$ have from 1.5 to $4\,\upmu \hbox {m}$ reduction in width compared to the dimension expected based on the design file (Fig. 3b; Table 1). Some of the deviations in width between the actual design and the obtained PLL-FITC spots can also be explained by alternative mechanisms. PDMS is known to shrink when cured^36,37^, and this shrinking may contribute to the differences observed. Recently Khadpekar et al showed that the width of the imprinted spots also depends on the weight applied onto the stamp during $\upmu \hbox {CP}$^38^. Adding a weight onto the stamps during stamping is expected to improve the transfer of the chemical from the PDMS pillar to the surface. In the current study a weight of 100 g was used, since this in previous results obtained in our laboratory was identified as optimal^18^. Furthermore PDMS surface wettability is also known to influence on the width of the imprinted surface spot^39^.

Cell line models are used in laboratories to study numerous physiological as well as pathophysiological processes. Data obtained from studies of cell line models are essential in order to identify therapeutic efficacy associated with drug response. Human umbilical vein endothelial cells (HUVECs) are used as a laboratory model system for the study of the function and pathology of endothelial cells (e.g., angiogenesis)^40^. Adeno-associated virus (AAV) AAV-293 cells are Human embryonic kidney 293 cells (HEK293) optimised for packaging of AAV virions and support high levels of viral protein expression^41^ Studies performed on cancer-derived cell lines like U87 (Glioblastoma^42^), OHS (Osteosarcoma^43^), SW480 (Colon^44^), PC3 (Prostate^45^), Hep-G2 (Liver^46^), and AY-27 (Bladder^47^), enable researchers to test hypotheses and thus improve the efficacy of cancer treatments.

When developing cellular arrays, it is essential to have a surface that resists non-specific adsorption of cells. The cytophilic areas will then be introduced on this cytophobic surface. PEG is a known passivizing agent for cells, and if coupled to PLL it can be immobilised onto glass surfaces through electrostatic interactions between the positively charged PLL and the glass. PEI and Matrigel are known to adhere cells. PEI is a cationic polymer, which forms interactions with the negatively charged surface of cells. Matrigel is a protein mixture that resembles the complex extracellular environment found in many tissues and is commonly used as a basement membrane matrix for culturing cells. It promotes cell adhesion primarily through their extracellular matrix (ECM) components laminin and collagen^48^. Cells immobilised using Matrigel demonstrate complex cellular behaviour, which is otherwise difficult to observe under laboratory conditions^48,49^. PEI is an alternative to Matrigel in the sense that PEI can also be used to immobilise mammalian cells. However, PEI differs from Matrigel by being cytotoxic, and it can induce both membrane damage and initiate apoptosis in mammalian cells^50,51^.

A number of factors influences on the quality of the SCA obtained. These factors include the properties of the cytophilic chemical, the size and surface properties of the cells that are to be immobilised, the size of the cytophilic spots as well as the separation distance between them, and cell load. Based on results obtained in the current study (Fig. 4c) we conclude that the width of the cytophilic spots should be approximately half of the cell diameter (Fig. 4b). If the cytophilic spot is $\ge$ cell size, the probability of obtaining multiple cells per spot increases (Fig. 4b). Moreover, the spacing between consecutive spots should be at least twice the cell size in order to obtain clear separation of cells on an array. As the spacing decreases the probability of obtaining bridges of cells between adjacent cytophilic surface spots on the array increases ($7\,\upmu \hbox {m}/7\,\upmu \hbox {m}$ in Fig. 4c). The cell load, defined as the density of cells in the cell suspension that is added to the functionalised surfaces, also influences on the quality of the SCAs. A low cell load leads to high probability of obtaining empty spots on the array but decreases the probability of obtaining multiple cells per spot. The cell load thus has the opposite effect of spot size: For large spot sizes compared to the cell size a low cell load may reduce the tendency for the unwanted event of obtaining multiple cells per spot (Fig. 4c). For small spot sizes compared to the cell size a high cell load may counteract the tendency for empty spots.

Both *E. coli* and *Synechococcus* cells were successfully immobilised onto PEI coated surface spots (Fig. 5). To our knowledge, this is the first documented fabrication of SCAs of unmodified *E. coli*. Regular arrays of *E. coli* have been obtained using $\upmu \hbox {CP}$ also in a previous study but this procedure required biotinylation of the cells prior to immobilisation^52^. In a previous study by Arnfinnsdottir and co-workers polydopamine (PD) was successfully used to immobilise unmodified *Pseudomonas putida* on an array^18^. However, PD does not support immobilisation of *C. reinhardtii*, *E. coli*, and *Synechococcus* cells (Supplementary Figure S12). This suggests that the surface properties of cells play a critical role in immobilisation of cells and the choice of cytophilic chemical must be optimised for each cell type. When fabricating SCAs, it is also essential to obtain a high probability for immobilisation of a single cell per cytophilic spot. As seen in Fig. 5, most of the cytophilic spots introduced by $7\,\upmu \hbox {m}\,/14\,\upmu \hbox {m}$ pillars were occupied by single *E. coli* cells. The optical density of the cell suspension used when obtaining these arrays was equal to 0.4. However, some cytophilic spots were occupied by either no cells or more than one cell. When immobilising small, non-spherical cells like *E. coli* this is challenging to avoid. The width of *Synechococcus* cells is 2.5 times smaller than the adhesive cytophilic spots, which resulted in multiple cells per spot. This challenge could have been overcome by reducing the size of the cytophilic spots. This would require changes in the photolithography process. However, when the *Synechococcus* cell density was reduced to 25% of the initial density, single cells could be obtained on the array. This is a significantly less time consuming approach to reduce the multiplicity of cells per cytophilic spot. The above observations suggest that both cell size, cell load, and width of cytophilic spot influences on the probability of obtaining single cells on the cytophilic spots.

The mammalian cells included in this study are above $15\,\upmu \hbox {m}$ in width. We therefore selected $15\,\upmu \hbox {m}/50\,\upmu \hbox {m}$ pillars to immobilise mammalian cells using Matrigel. The preparation of a single-cell suspension is a crucial step for obtaining SCAs of mammalian cells. A common method to obtain single-cell suspensions from cultured cell lines is trypsin digestion which helps cells to detach from the adherent substrate and which leads to single cells instead of confluent cells. The cells should be exposed to trypsin for at least 1–3 min prior to immobilisation. This procedure is expected to yield a cell population with high viability, minimal cell debris or aggregates, and preserved cell surface antigens. However, even after trypsin treatment, some cell aggregates might be present in the cell suspension. This might explain the presence of cell aggregates instead of single cells on some of the cytophilic spots on the array. Also, cell adhesion strength is controlled by inter-molecular spacing of adhesion receptors^53^. Each cell type has different amounts of adhering receptors. Cells weakly adhering to Matrigel can be easily washed off during immobilisation. This can be one of the reasons why some of the cytophilic spots do not contain any immobilised cells. If the interaction between the cells and the cytophilic spots is weak it is essential that the surfaces are handled gently during cell immobilisation and subsequent washing steps.

The fact that multiple soft lithography stamps can be produced from the same master is well known. However, the PDMS stamps used for $\upmu \hbox {CP}$ can also be reused multiple times as long as the pillars on the stamp are intact. SEM images of PDMS stamps used 1 time show that the pillars are intact (Supplementary Figure S13). This robustness of the PDMS stamps is important from an economical perspective where cost-effective and reliable techniques are required to study single-cell biology. Altogether, our study documents the importance of a range of factors that influences on the $\upmu \hbox {CP}$ methodology when used for the fabrication of SCAs. Furthermore, the study underpins the importance of documenting these critical parameters. The dimensions defined in the design file used in the fabrication of the master as well as the choice of photoresist and the exposure energy used for fabrication, together determine the size of the obtained cytophilic surface spots. It is also demonstrated that in order to fabricate an SCA, both the cytophobic and the cytophilic chemical must be carefully chosen to fit with the properties of the cells that are to be immobilised. Additionally, the width of the cytophilic surface spot, the spacing between these spots as well as the cell load used are important factors that should each be optimised for the cells in question. Only when all of these factors are carefully optimised SCAs can be obtained.

## Methods

### Cell cultures

All chemicals, unless otherwise stated, were purchase from Sigma-Aldrich. Dulbecco’s Modified Eagle Medium (DMEM), Roswell Park Memorial Institute (RPMI) 1640, Minimal Essential medium Eagle (M4655) and Non-Essential Amino Acids Solution (NEAA) were purchased from Thermo Fisher Scientific. The chemicals were used as received.

*Chlamydomonas reinhardtii* CC-5155, a cw15 cell-wall deficient mutant, was obtained from the Chlamydomonas Resource Center (https://www.chlamycollection.org/). Cells were grown mixotrophically in Tris-acetate-Phosphate (TAP) media in canonical glass flasks at $25\,^{\circ }\hbox {C}$, with an agitation of 175 rpm and with a light intensity of $40\ \upmu \hbox {mol}$ photons $\hbox {s}^{-1}\,\hbox {m}^{-2}$. Cells were harvested when $\hbox {OD}_{730}$ reached 2.

*Escherichia coli* DH5$\alpha$ cells containing the plasmid pHH100, harbouring the mCherry gene, were grown at $37 \,^{\circ }\hbox {C}$, 225 rpm in Lysogeny Broth (LB) media (10 g/L tryptone (OXOID), 5 g/L yeast extract (OXOID) and 5 g/L NaCl (VWR) containing kanamycin (50 mg/L,PacReac AppliChem). Cells were harvested when $\hbox {OD}_{600}$ reached 0.4.

*Synechococcus* sp. PCC 7002 cells were cultured in $$\hbox {AA}^{+}$$-medium at $$30\,^{\circ }\hbox {C}$$ under constant illumination of $$30\,\upmu \hbox {E\,m}^{-2}\,\hbox {s}^{-1}$$ in batches of 30 mL in Nunc Non-treated T75 EasyFlasks with filter caps. AA+-medium is a derivative of A+^54^ with the P1 trace metal solution replaced by a 1000x BG-11 trace mineral solution^55^. The exact composition of the medium is (per liter): 18 g NaCl, 0.6 g KCl, 1.0 g $$\hbox {NaNO}_3$$, 5.0 g $$\hbox {MgSO}_4$$$$\cdot$$ 7$$\hbox {H}_2$$O, 50 mg $$\hbox {KH}_2\hbox {PO}_4$$, 266 mg $$\hbox {CaCl}_2$$, 30 mg $$\hbox {Na}_2$$EDTA $$\cdot$$ 2$$\hbox {H}_2$$O, 3.89 mg $$\hbox {FeCl}_3$$$$\cdot$$ 6$$\hbox {H}_2$$O, $$4\,\upmu \hbox {g}$$ vitamin B12, 10 mL 0.825M Tris/HCl (pH 8.2), 1 mL 1000x BG11 trace mineral solution. The 1000x BG11 trace mineral solution contains $$\hbox {2.860 g/L }\hbox {H}_3\hbox {BO}_3$$, $$\hbox {1.810 g/L }\hbox {MnCl}_2$$$$\cdot \ 4\hbox {H}_2\hbox {O}$$, $$\hbox {0.222 g/L }\hbox {ZnSO}_4\,\cdot \,7\hbox {H}_2\hbox {O}$$, $$\hbox {0.390 g/L}\hbox {Na}_2\hbox {MoO}_4\,\cdot \,2\hbox {H}_2\hbox {O}$$, $$\hbox {0.079 g/L }\hbox {CuSO}_4\,\cdot \,5\hbox {H}_2\hbox {O}$$ and 0.0494 g/L Co ($$\hbox {NO}_3)\ 2\ \cdot \,6\hbox {H}_2\hbox {O}$$. The cells were harvested for further studies when $$\hbox {OD}_{730}$$ was equal to 0.4.

HUVEC cells were cultured in Endothelial Cell Growth Medium-2 (EGM-2) BulletKit (Lonza). AAV-293 cells were cultured in Dulbecco’s Modified Eagle Medium (DMEM) supplemented with 10% fetal bovine serum (FBS) (Thermo Fisher Scientific), 1% (v/v)penicillin-streptomycin (Thermo Fisher Scientific), 1% (v/v) MEM Non-Essential Amino Acids Solution and 1 mM Sodium Pyruvate (Thermo Fisher Scientific). U87MG cells were cultured in DMEM supplemented with 2 mM glutamine (Biochrome), $100\,\upmu \hbox {g/mL}$ gentamicin, and $2.5\,\upmu \hbox {g/mL}$ amphotericin. OHS cells were cultured in Roswell Park Memorial Institute 1640 media (RPMI) supplemented with 10% FBS, 0,5%L-glutamine and 1% penicillin-streptomycin. PC3 cells were cultured in high glucose DMEM without sodium pyruvate (Gibco) supplemented with 10% FBS and without antibiotics. SW480 cells were cultured in RPMI supplemented with 2 mM glutamine (Biochrome), $100\,\upmu \hbox {g/mL}$ gentamicin, and $2.5\,\upmu \hbox {g/mL}$ amphotericin. Hep-G2 cells were cultured in Low glucose DMEM (Gibco) supplemented with 10% FBS, 2 mM L-glutamine, 1x MEM, Non-Essential Amino Acids Solution (NEAA), 1 mM sodium pyruvate and 100 U/mL penicillin-streptomycin. AY-27 cells were cultured in RPMI supplemented with 10% FBS, 0.34% L-glutamine and 1% penicillin-streptomycin. All cultures were maintained at $37\,^{\circ }\hbox {C}$ in a humidified atmosphere of 5% $\hbox {CO}_2$. When the cells were sub confluent they were trypsinized. Trypsinization of the cells was carried out using 1x trypsin-EDTA solution, the cells were collected by spinning at 1500 rpm for 5 min at room temperature.

### Design files

The designs used to fabricated the PDMS stamps used for both dose test experiments as well as the fabrication of the single-cell arrays (Fig. 1a) were designed using CleWin Layout Editor (Version 4.3.5.0). Design (1) consisted of a series of squares of increasing size, ranging from $1\,\upmu \hbox {m}$ to $10\,\upmu \hbox {m}$ width, separated by $10\,\upmu \hbox {m}$. According to the nomenclature system used throughout this paper, these designs will be referred to as $1\,\upmu \hbox {m}$-$10\,\upmu \hbox {m}/10\,\upmu \hbox {m}$. Design (2) consisted of squares with a side width equal to $7\,\upmu \hbox {m}$ separated by either 7 $\upmu \hbox {m}$, $14\,\upmu \hbox {m}$ or $50\,\upmu \hbox {m}$. These designs are referred to as 7 $\upmu \hbox {m}/7\,\upmu \hbox {m}$, $7\,\upmu \hbox {m}/14\,\upmu \hbox {m}$ and $7\,\upmu \hbox {m}/50\,\upmu \hbox {m}$, respectively. Design (3) consisted of squares with a side width equal of either $15\,\upmu \hbox {m}$, $20\,\upmu \hbox {m}$, $25\,\upmu \hbox {m}$ or $30\,\upmu \hbox {m}$ width, separated by $50\,\upmu \hbox {m}$. They are referred to as $15\,\upmu \hbox {m}/50\,\upmu \hbox {m}$, $20\,\upmu \hbox {m}/50\,\upmu \hbox {m}$, $25\,\upmu \hbox {m}/50\,\upmu \hbox {m}$, $30\,\upmu \hbox {m}/50\,\upmu \hbox {m}$, respectively. All the designs were replicated multiple times to form a square shaped array with a width equal to approximately 2-3 mm.

### Photolithography

The photolithography was performed using standard maskless lithography. A 2-inch silicon wafer was used to fabricate design 1, a 4-inch silicon wafer was used to fabricate design 2 and 3. Prior to use the wafers were first washed with acetone followed by isopropanol (IPA) and finally dried using nitrogen gas. The dried wafer was ozone treated (Novascan) for 3 min, followed by dehydration bake at $180\,^{\circ }\hbox {C}$ for 20 min. The dehydrated wafers were spin coated for 33 s at 3000 rpm with the acceleration of 300 rpm. For design 1 and 2 the negative photoresist mr-DWL5 was used whereas for design 3 the negative photoresist mr-DWL40 was used (micro resist technology GmbH, Germany). Soft baking of the resist was obtained by gradually increasing the temperature of the hot plate from $50 \,^{\circ }\hbox {C}$ to $90 \,^{\circ }\hbox {C}$. The mr-DWL5 resist was baked for 2 min while mr-DWL40 was baked for 10 min. The soft baked resist layers were allowed to gradually cool down on the hot plate by decreasing the temperature to $50 \,^{\circ }\hbox {C}$. This step was followed by a relaxation time of 10 min for mr-DWL5 and 1 h for mr-DWL40 at room temperature. The resist-coated wafers were exposed to UV light (UV 405 nm) using MLA 150 (Maskless Aligner 150, Heidelberg Instruments, Germany) to directly transfer the design to the resists. A dose test was performed in order to identify the optimal exposure energy for each of the features included in design 1. The lowest and highest exposure energies were set to 150 mJ/$\hbox {cm}^2$ and 365 mJ/$\hbox {cm}^2$ respectively. Within this interval of exposure energies, the energy was increased stepwise, with 15 mJ/$\hbox {cm}^2$ per step. Based on the results obtained in this dose test, the optimal exposure energies when working with design 2 and 3 was chosen. For design 2 and 3 the exposure energy was set to 190 mJ/$\hbox {cm}^2$ and 500 mJ/$\hbox {cm}^2$, respectively. The post exposure bake was performed using a procedure that resembled the one used for the soft bake. The relaxation time after post exposure bake was set to 1 h for mr-DWL5 and 2 h for mr-DWL40 at room temperature. The resist were developed after addition of the developper mr-Dev 600 (micro resist technology GmbH, Germany). The development was allowed to proceed for 2 min for mr-DWL5 and 6 min for mr-DWL40 under constant stirring. The developed wafers were thoroughly washed in IPA and dried using nitrogen gas. The silicon wafer fabricated by photolithography is now called “master” and is further used for soft lithography. Post development inspection was performed on each master using brighfield (BF) top down light with 20$\times$ magnification (Nikon, Eclipse LV150).

### Soft lithography

The masters fabricated by photolithography were silanized using a fluorosilane (1H,1H,2H,2H-perfluorootyl(trichlorosilane)) for 1 h in a vacuum chamber. The aim of the silalization was to avoid adhesion of the polydimethyl siloxane (PDMS, Dow $\hbox {Corning}^{\circledR }$) to the surface of the master at later stages in the process. A mixture containing 90% PDMS and 10% wt initiator (Sylgard 184 kit, Dow $\hbox {Corning}^{\circledR }$) was thoroughly mixed for 5 min and degassed for 20 min. The degassed solution was casted onto the silanized master in a petri dish and baked for 3 h at $65 \,^{\circ }\hbox {C}$. The PDMS was subsequently peeled off from the master. Some of the PDMS surfaces were imaged using SEM. The PDMS surfaces were used for microcontact printing ($\upmu \hbox {CP}$) following the procedure described below.

### Scanning electron microscopy

A FEI Helios NanoLab DualBeam FIB (Focused Ion Beam) was used to record electron micrographs. The ion beam was not used for the presented data. The electron beam acceleration voltage was set to 3 kV unless otherwise indicated. Micrographs were generated using secondary electrons, detected using an ICE-detector. A sample tilt equal to $45\,^{\circ }$ was used. The samples were coated with 5 nm Pt/Pd (80/20) prior to imaging, applied using a Cressington 208 HR B sputter coater.

### Fluorescein isothiocyanate (FITC) staining of PDMS stamps

In order to investigate the performance of the fabricated PDMS stamps, they were used for deposition of PLL-FITC ($\hbox {M}_W$ 30 000–70 000) onto glass surfaces. PDMS stamps made based on design 1, 2 or 3 were all cowered with a solution containing PLL-FITC (0.5 mg/mL) for 30 min and blow dried using a stream of nitrogen gas. The PLL-FITC coated stamps thus obtained were placed pattern-side down on a clean glass slide and left for 30 min with a 100 g weight on top in order to ensure optimal contact between the stamp and the glass surface. To avoid photobleaching of the fluorophore the procedure was performed in the dark. The PDMS stamps were then carefully removed from the glass surface, leaving an array of PLL-FITC coated spots on the surface. These imprinted surfaces were inspected using fluorescence microscopy in order to determine the shape and size of the PLL-FITC coated spots.

### ($\upmu \hbox {CP}$) based deposition of $\upmu$-sized spots of PEI and Matrigel

In order to obtain cellular microarrays, cytophilic chemicals were deposited using $$\upmu \hbox {CP}$$ onto glass slides precoated by cytophobic chemicals, as previously described by us^18^. In order to immobilise the cells described in the current paper, the cytophilic chemicals PEI and Matrigel were used. Micron-sized surface spots were introduced through $$\upmu \hbox {CP}$$ based deposition of these chemicals on glass surfaces passivated through coating with the cytophobic chemical PEG. The surface modifications were carried out in the following way. Wilco dishes were first assembled according to the specification by the manufacturer. The glass slide were rinsed with 70% ethanol followed by MilliQ water and blow-dried with nitrogen gas. The glass slide were coated with 0.1 mg/mL PLL (20 kDa)-g-PEG (2 kDa) buffered in 10 mM HEPES, pH 7.4 solution for 60 min. The slides were subsequently rinsed in MilliQ water and dried with nitrogen gas. PDMS stamps were used to introduce patterns of polyethyleneimine (PEI, Mw 750 000 by LS, 50% wt in $$\hbox {H}_2$$O) and Matrigel ($$\hbox {Corning}^{\circledR }$$$$\hbox {Matrigel}^{\circledR }$$ Basement Membrane Matrix, Phenol Red-free). PDMS stamps were incubated with either aqueous 1 % wt PEI or 1 mg/mL of Matrigel for 60 min at room temperature and on ice, respectively. The stamps were then blow dried using a stream of nitrogen gas and placed pattern-side down on the PEGylated glass slides for 60 min with a 100 g weight on top. The Matrigel coated PDMS stamps were incubated at $$37\,^{\circ }\hbox {C}$$ to effectively transfer the protein components. The PDMS stamps were then carefully removed from the glass surface, leaving the PEI and Matrigel surface spots arranged in an array as dictated by the design used.

### *C. reinhardtii*, *E. coli* and *Synechococcus* cell immobilisation using PEI

In order to immobilise *C. reinhardtii* cells, $500\,\upmu \hbox {L}$ of cell suspension, containing cells in the late stationary phase, was placed on PEI imprinted surfaces. The surfaces were imprinted using design 2 and 3, and the PDMS stamps had been left in contact with the glass surface for 5 min. A $500\,\upmu \hbox {L}$ of log phase ($\hbox {OD}_{730} = 0.4$) *E. coli* and *Synechococcus* cells were immobilised as above using PDMS stamp feature size of $7\upmu \hbox {m}/14\upmu \hbox {m}$ spacing. In order to investigate the effect of the cell load, *C. reinhardtii* and *Synechococcus* cell cultures were diluted 5 times prior to additin onto imprinted surfaces. Unattached cells were removed by gentle flushing with culture media. The arrayed surface was immediately covered with culture medium and imaged using light microscopy.

### Mammalian cell immobilisation using Matrigel

The mammalian cells were counted, and diluted to the concentration of 1 million cells per millilitre of sterile media without serum. If the cells were observed to be confluent, they were exposed to trypsin digestion prior to counting. Cells were mixed with live-dead staining kit of Invitrogen (containing Calcein AM (4 mM) and Ethidium homodimer-1 (2 mM)). $15\upmu \hbox {m}/50\upmu \hbox {m}$ PDMS stamps from design 3 was used to deposit Matrigel in a predefined pattern on pegylated glass surfaces. $500\,\upmu \hbox {L}$ of a solution containing stained cells were placed on the Matrigel imprinted surface. The surfaces were left for 60 min in a standard tissue culture incubator at $37 \,^{\circ }\hbox {C}$, 100% humidity, 95% air, and 5% $\hbox {CO}_2$. Subsequently, unattached cells were removed from the array by gentle flushing with culture medium. The arrayed surface was immediately covered with culture medium and further studied using light microscopy.

### Fluorescence microscopy

An inverted microscope (Axio Observer.Z1 from Zeiss, 2.3.64.0) with 20x air objective (NA 0.8) was used for image acquisition. FITC, mCherry, calcein AM, ethidium homodimer and 5-CNF filters were used to inspect the imprint of the PLL-FITC coated stamps as well as the viability of the immobilised cells. Initial image processing was performed using the Zeiss image analysis software (2.3.64.0).

### Image processing

Image processing such as adjusting contrast/brightness and cropping was conducted using Fiji, ImageJ v. 2.0.0-rc-69/1.52q^56^. All scale bars are added as an overlay using ti*k*z. SEM micrographs had brightness/contrast adjusted manually, and instrument information bar at image bottom was removed. Where images are rotated to better present the data (Fig. 2, Supplementary Figure S1) bilinear interpolation was used. For estimates of spot size image processing and subsequent analysis was performed using FIJI and R. Image processing in Fiji was conducted as follows using built-in functionality: An automatic adjustment of brightness and contrast was performed. Once brightness and contrast were adjusted images were de-speckled and thresholded using the Max Entropy algorithm and default settings. Thresholded micrographs were further analysed using the built-in function “Analyze Particles”. Circularity and area filters were set on an image-by-image basis to include only feature imprints. In rare cases where known contaminants were not automatically removed using automated settings these were manually removed from the dataset before processing. A bounding box (rectangle) was drawn around each spot, the width of this box was measured and is reported throughout the work as a metric describing spot size. Results were exported from FIJI as csv files and further processed in R v. 3.5.0^57^. Data was plotted using ggplot2^58^, exported as Ti*k*Z code and rendered using LaTeX.

## Supplementary information

Supplementary information.
