# Supplementary material for: Effect of design geometry, exposure energy, cytophilic molecules, cell type and load in fabrication of single-cell arrays using micro-contact printing
Source: Sci Rep. 2020 Sep 16;10:15213. doi: 10.1038/s41598-020-72080-w (PMC7494944; doi:10.1038/s41598-020-72080-w)
Supplement: Supplementary file 1 — Supplementary information. [file 41598_2020_72080_MOESM1_ESM.pdf]

Effect of design geometry, exposure energy, cytophilic molecules, cell type and load in fabrication of single-cell arrays using micro-contact printing

Swapnil Vilas Bhujbal[1+], Maren Dekov[1+], Vegar Ottesen[2], Karen Dunker[1], Rahmi Lale[1], and Marit Sletmoen[1*]

[1]Department of Biotechnology, Norwegian University of Science and Technology, NO-7491 Trondheim, Norway

[2]Department of Chemical Engineering, Norwegian University of Science and Technology, NO-7491 Trondheim, Norway

[+]these authors contributed equally to this work.

[*]Corresponding author: marit.sletmoen@ntnu.no

Supplementary Data

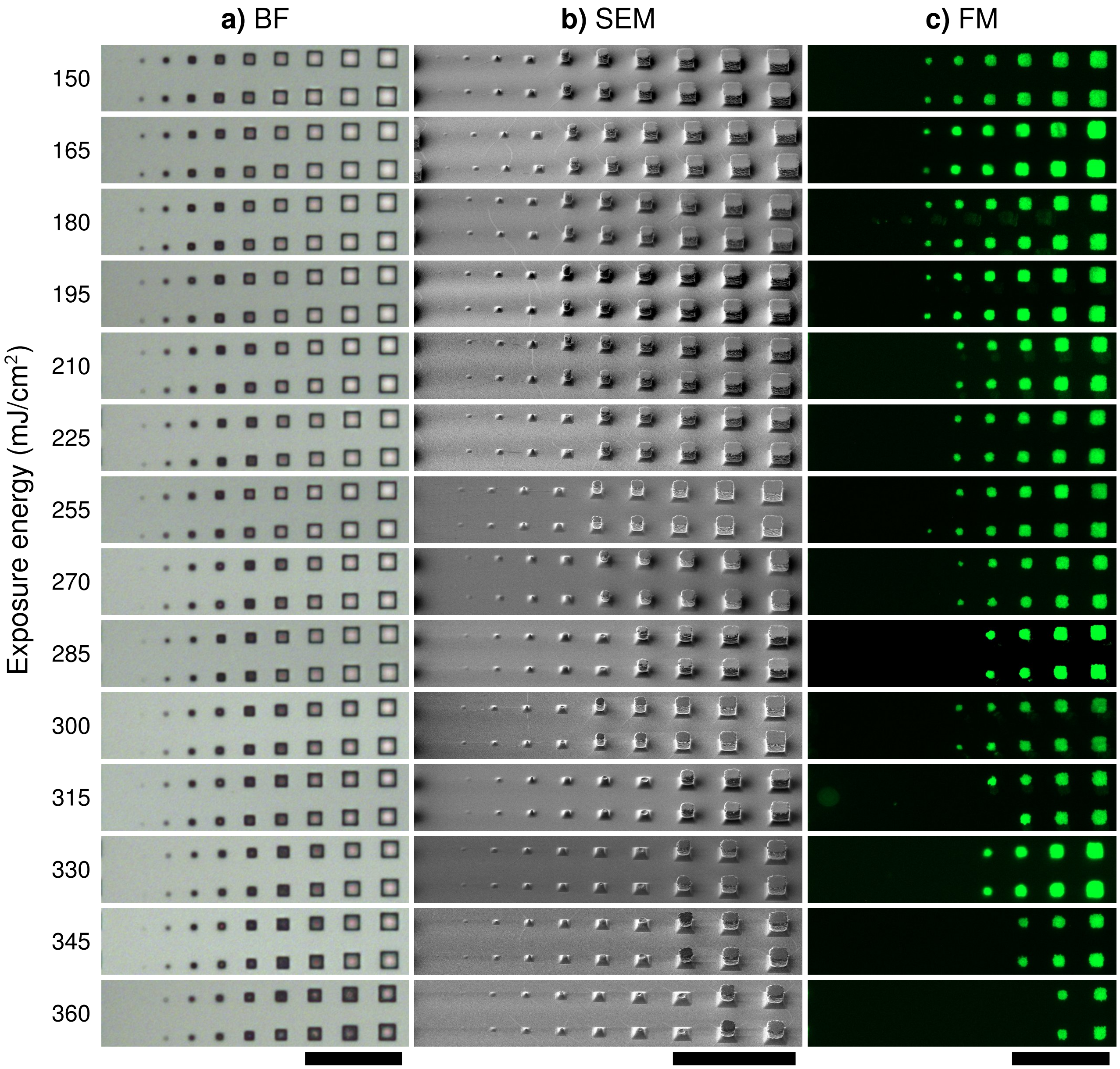

Supplementary Figure 1. Relationship between exposure energy and different sized square features (1- 10 µm) with 10 µm horizontal spacing using mr-DWL5 photoresist. (a) Light microscope images of exposed photoresist on a silicon substrate, at different exposure energy. Rounded areas indicate overexposure of the photoresist. Square-shaped areas imply that the exposure was effective. Images were taken at 20x magnification. (b) SEM images of the PDMS stamps at different exposure energy. Features below 5 µm are not obtained in any of the tested exposure energies. Increasing the exposure energy resulted in broken features for smaller feature width which were barely visible and with distorted squares. SEM magnification was set to 800x. (c) FM images of surfaces patterned using FITC stained PDMS stamps shown for different exposure energies. Overexposed areas are not visible in the FITC staining of the PDMS stamps. Images were taken at 20x magnification. The scale bar is shown as a black line below each column of micrographs. All scale bars represent a distance equal to 50 µm long. Feature size decreases with increasing exposure energy. Smaller feature sizes were rounded compared to larger sized features.

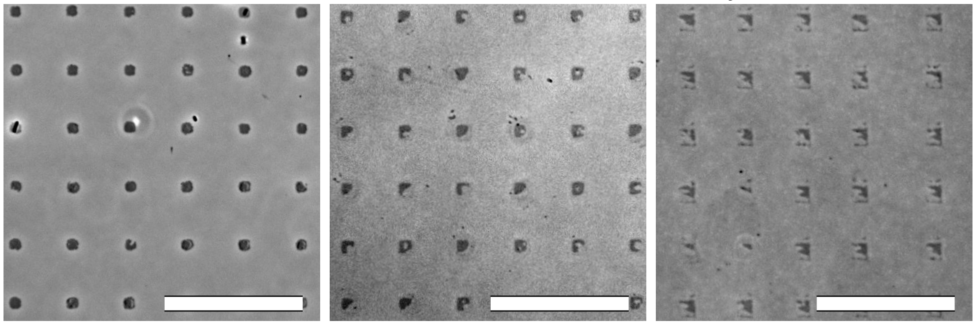

Supplementary Figure 2. Polydopamine does not support immobilisation of *E.coli*, *C. reinhardtii* and *Synechococcus* cells. The spot width is of 7 µm/14 µm. Scale bars are 50 µm long.

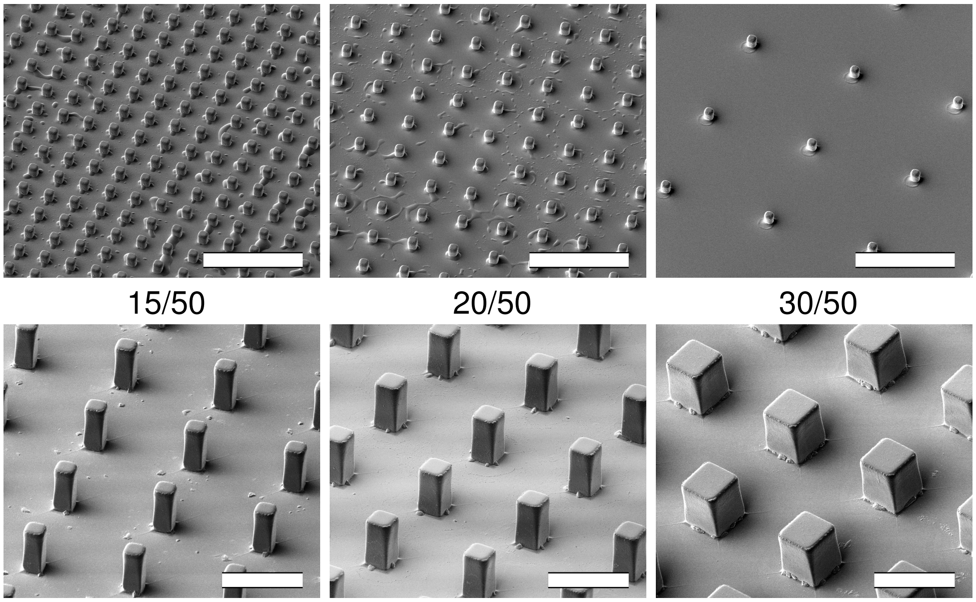

Supplementary Figure 3. SEM images of the 1 time used PDMS stamp. The features on the PDMS stamps are intact.All values are in µm. Scale bars are 50µm long.
